# Determination of brain death using ^99m^Tc-HMPAO scintigraphy and transcranial duplex sonography in a patient on veno-arterial ECMO

**DOI:** 10.1186/s42466-023-00298-w

**Published:** 2024-01-25

**Authors:** Albrecht Günther, Anke Werner, Michael Fritzenwanger, Martin Brauer, Martin Freesmeyer, P. Christian Schulze, Farid Salih, Robert Drescher

**Affiliations:** 1 Klinik für Neurologie, Universitätsklinikum Jena, Am Klinikum 1, 07747 Jena, Germany; 2 Klinik für Nuklearmedizin, Universitätsklinikum Jena, Jena, Germany; 3 Klinik für Innere Medizin 1, Universitätsklinikum Jena, Jena, Germany; 4 Klinik für Anästhesiologie und Intensivmedizin, Universitätsklinikum Jena, Jena, Germany; 5 Klinik für Neurologie mit Experimenteller Neurologie, Charitè Universitätsmedizin, Berlin, Germany

Patients suffering circulatory arrest (CA) may require veno-arterial extracorporeal membrane oxygenation (va-ECMO) when unresponsive to conventional cardiopulmonary resuscitation (CPR) in order to maintain systemic circulation. Despite this intervention, some of these patients develop irreversible loss of whole brain function (i.e., brain death, BD) due to hypoxic-ischemic encephalopathy or an intracranial hemorrhagic complication. Apart from the clinical examination, ancillary tests are used to eventually determine the patient’s brain death [1].

According to the latest update of the German Medical Association (GMA) guideline for the determination of BD, effective from September 2022, in va-ECMO patients ancillary tests are restricted to electrophysiological methods due to lack of evidence for radiological / perfusion techniques [2]. Here we report a case in whom BD was determined according to the current guidelines. In addition, cerebral perfusion was investigated with ^99m^Tc-HMPAO scintigraphy and transcranial Duplex-sonography, respectively.

## Patient and methods

**Fig. 1:**
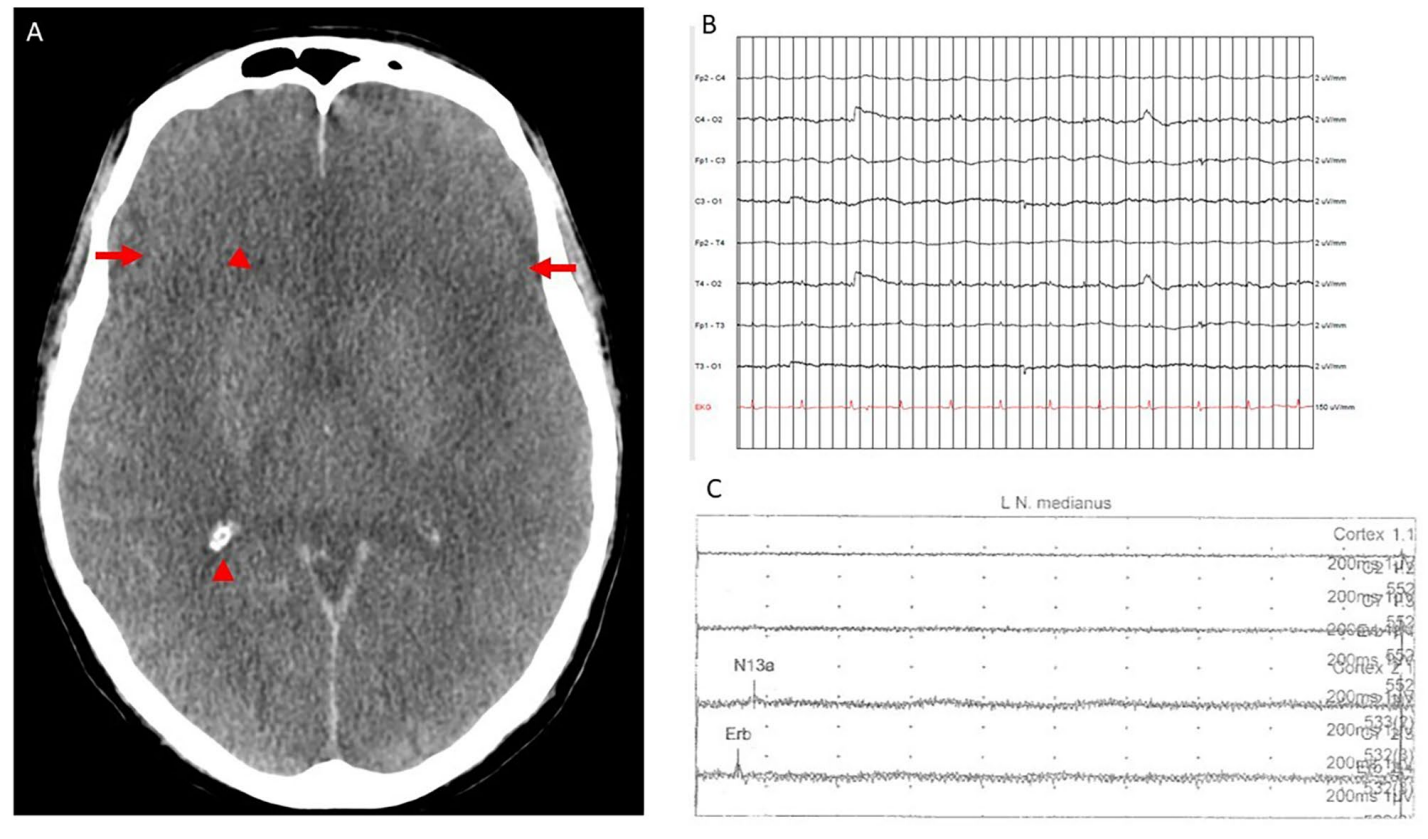
Non-contrast computer tomography (CT) of the brain **(A)** four days after the event shows generalized cerebral edema with complete effacement of sulci (arrows) and ventricles (arrowheads). Flatline EEG at 7µV **(B)**. Sensory evoked potential of median nerve (**C**, left side; right side not shown) revealed bilateral loss of cortical N20

**Fig. 2:**
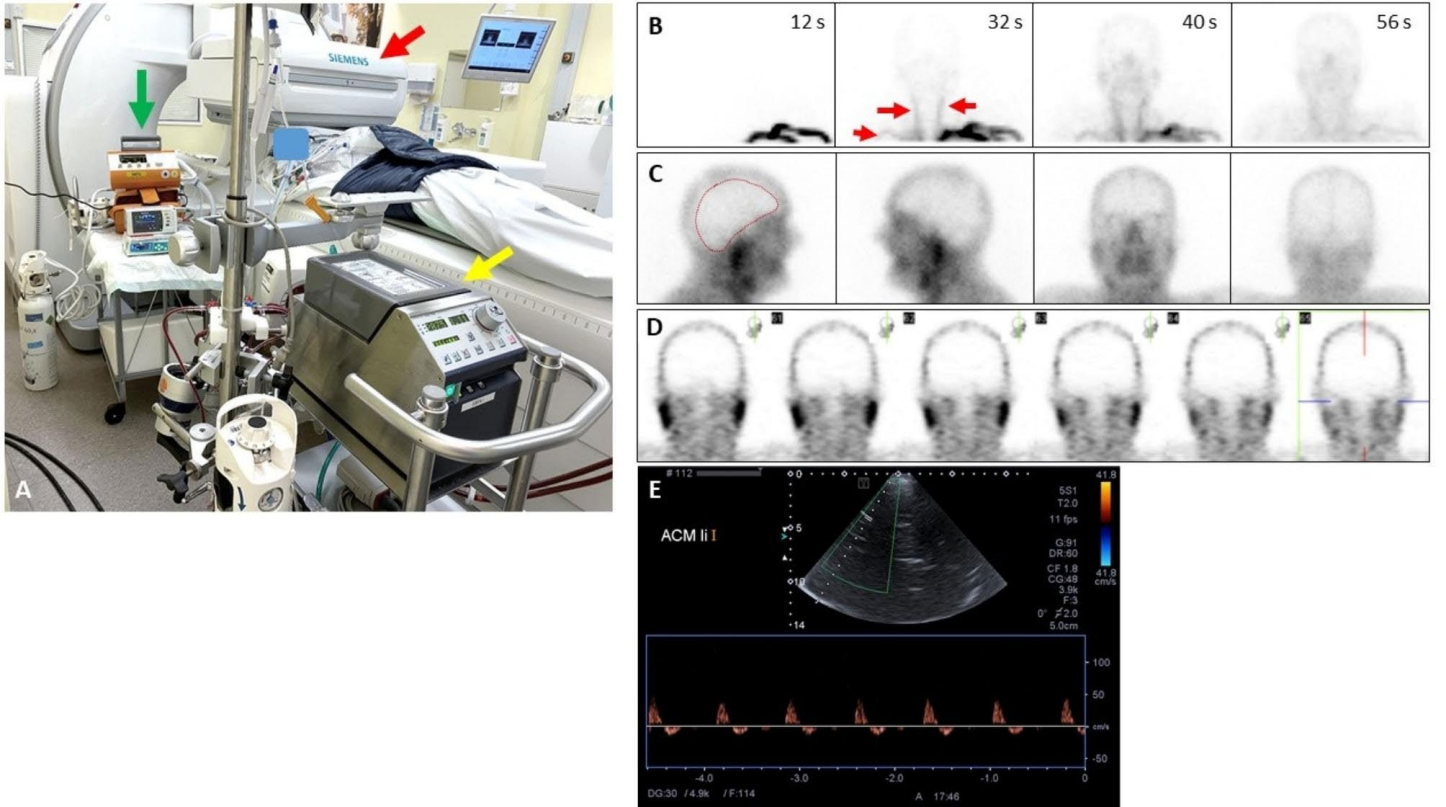
Imaging setup for perfusion scintigraphy with ^99m^Tc-HMPAO of the patient on vaECMO **(A)**, with gamma camera (red arrow), portable respirator (green arrow) and ECMO pump/oxygenator (yellow arrow). The dynamic acquisition **(B)** shows inflow of the tracer from the left arm (12s after injection), appearing in the carotid and subclavian arteries 32s after tracer injection (red arrows) and in the extraaxial soft tissues of the head. Static planar images acquired after 30 min **(C)** do not show tracer uptake in the brain (dotted line), which can be confirmed by tomographic SPECT imaging (**D**, examplary coronal views). Transcranial Duplex-sonography **(E)**. Circulation arrest in the left middle cerebral artery (MCA). Right MCA, distal carotid artery, bilateral V4 segments and basilar artery showed the same pattern

A 58-years-old man suffered an in-hospital cardiac arrest due to asystolia. Cardio-pulmonary resuscitation was successful, and vaECMO was established. However, severe hypoxic-ischemic encephalopathy developed (Fig. 1A), resulting in BD four days after admission. BD was determined and confirmed by clinical criteria, electroencephalography (EEG; Fig. 1B) and somatosensory evoked potentials (Fig. 1C) according to the GMA guideline requirements [2]. With regard to apnea testing (AT) a simultaneous arterial blood gas analyses (ABG) were taken from right radial artery and postoxygenator ECMO circuit following the GMA guidelines. Due to minor differences in ABG values for p_a_CO_2_-levels between both sample sites it took 34 min until both analyses reached the required cut-off level at the end of AT. However, all clinical findings including AT were compatible with BD. To provide more evidence for radiological techniques in BD determination for patients with va-ECMO we studied cerebral perfusion by color-coded duplex sonography and brain perfusion scintigraphy (BPS). For technical details of the ^99m^Tc-HMPAO scintigraphy see supplementary material and Fig. 2A. BPS showed a complete loss of brain perfusion (Fig. 2B-D). Sonographically, biphasic flow with equal areas under the curve was seen in the cerebral arteries (example: left middle cerebral artery (MCA), Fig. 2E; same patterns were detected in the right MCA, distal internal carotid and posterior cerebral arteries, V4 segments of vertebral arteries, and basilar artery) corresponding to a residual cardiac output in our patient. An organ donation was not realized according to the patient‘s will.

Scintigraphic method: The patient was positioned supine in a SPECT/CT scanner (Symbia Intevo Bold, Siemens Healthineers, Erlangen, Germany) (Fig. 2A). 740 MBq ^99m^Tc–hexamethylpropyleneamine oxime (^99m^Tc-HMPAO) were injected intravenously in a 10 ml saline bolus through an 18G cannula at the left arm. Dynamic acquisition (anterior view, LEHR collimator, matrix 64 × 64, zoom 1.0) was started simultaneously with tracer injection, continued for 2 min (12 frames, 2 s per frame, followed by 12 frames, 8 s per frame), and confirmed tracer flow to the carotid arteries (Fig. 2B). From 30 min after tracer injection, anterior, posterior and lateral static images were acquired (anterior and posterior view, matrix 128 × 128, zoom 1.0, 5 min or 500000 counts) (Fig. 2C), followed by a SPECT of the head (32 angles, 20 s per angle; Fig. 2D). No tracer accumulation was seen in the brain tissue. Finally, a planar image of the upper abdomen was acquired and confirmed tracer stability (not shown; a high amount of ^99m^Tc not bound to HMPAO would accumulate in the stomach wall).

## Discussion

The present case addresses a methodological bottleneck in the determination of brain death, which has arisen as a result of the recently published GMA BD guideline [2]. There, it is concluded that due to a lack of scientific evidence, imaging procedures for the detection of cerebral circulatory arrest, including duplex sonography, CT-angiography as well as BPS, in patients undergoing va-ECMO should not be applied. Even though advantageous because of its bedside applicability sonographic ascertainment of intracranial pulsatile vascular flow signals might not be possible in many vaECMO patients due to low cardiac output [3].

The overall number of ECMO therapies has increased significantly, partly owed to the COVID-19 pandemic and, concomitantly, the number of nationwide BD diagnostic procedures that neurologists/neurointensivists are expected to perform in such patients. The methodological limitation imposed by the current guideline now confronts many BD diagnosticians with increasing challenges, even independent of the COVID pandemic, since in many ECMO patients apnea testing is complicated. Furthermore, electrophysiological methods maybe technically disturbed by artifacts.

Thus, investigating BPS could be valuable to increase the understanding and use of this method in BD determination. Two radiopharmaceuticals, [^99m^Tc]Tc-hexamethylpropylenamine oxime (HMPAO) and [^99m^Tc]Tc-ethylcisteinate dimer (ECD), are established and approved for brain perfusion scintigraphy and single-photon emission computed tomography (SPECT). Pathophysiologically, these lipophilic agents cross the blood-brain-barrier to perivascular astrocytes and neurons in proportion to the regional cerebral blood flood (CBF), and are converted to a hydrophilic compound resulting in intracellular trapping [4, 5]. The maximum intracellular retention of the radioactive tracer is reached during the first minute after injection. At least 85% remain nearly stable for up to 24 h after injection, which allows delayed imaging [6]. The same principle – taking a snapshot of tissue perfusion with a radioactive tracer and image it later - is used in scintigraphic myocardial perfusion imaging. In brain death, SPECT typically shows no cerebral uptake („hollow-skull“ or „empty skull“ sign), with persistant perfusion of the extraaxial soft tissues of the head.

Contrary to contrast-enhanced CT and MR angiography, brain perfusion scintigraphy does not depend on the passage of a short bolus of contrast media through the vasculature in seconds, but on the trapping of a radioactive tracer in perfused tissue. It has also been shown that brain perfusion scintigraphy with HMPAO tends to underestimate CBF in high-flow regions and to overestimate CBF in low-flow regions [7]. Therefore, perfusion scintigraphy may be particularly robust in patients with varying and possibly slow circulation/low pressure states, or under va-ECMO treatment. Taran et al. indicated that radiopharmacological kinetics are not substantially affected by ECMO and szintigraphic methods have been used successfully in ECMO patients [8].

We recommend further investigations of brain perfusion in patients on va-ECMO support to evaluate how circulatory changes by the ECMO pump in relation to the residual cardiac output may influence different brain perfusion imaging methods and to establish brain perfusion scintigraphy as well as duplex sonography as methods to diagnose circulatory arrest in clinically brain dead patients on va-ECMO support.

## Data Availability

The datasets of the current study available from the corresponding author on reasonable request.

## List of abbreviations

ABG: arterial blood gas
AT: apnea testing
BD: brain death
BPS: brain perfusion scintigraphy
CA: cardiac arrest
CBF: cerebral blood flood
CT: computer tomography
ECD: [^99m^Tc]Tc-ethylcisteinate dimer
EEG: electroencephalography
GMA: German Medical Association
HMPAO: [^99m^Tc]Tc-hexamethylpropylenamine oxime
MCA: middle cerebral artery
SPECT: single-photon emission computed tomography
va-ECMO: veno-arterial extracorporeal membrane oxygenation
